# Influence of Shell Thickness on the Colloidal Stability of Magnetic Core-Shell Particle Suspensions

**DOI:** 10.3389/fchem.2018.00201

**Published:** 2018-06-05

**Authors:** Frances Neville, Roberto Moreno-Atanasio

**Affiliations:** ^1^School of Environmental and Life Sciences, University of Newcastle, Callaghan, NSW, Australia; ^2^School of Engineering, University of Newcastle, Callaghan, NSW, Australia

**Keywords:** Discrete Element Method, computer simulations, core-shell particles, magnetic chains, electrical double layer

## Abstract

We present a Discrete Element study of the behavior of magnetic core-shell particles in which the properties of the core and the shell are explicitly defined. Particle cores were considered to be made of pure iron and thus possessed ferromagnetic properties, while particle shells were considered to be made of silica. Core sizes ranged between 0.5 and 4.0 μm with the actual particle size of the core-shell particles in the range between 0.6 and 21 μm. The magnetic cores were considered to have a magnetization of one tenth of the saturation magnetization of iron. This study aimed to understand how the thickness of the shell hinders the formation of particle chains. Chain formation was studied with different shell thicknesses and particle sizes in the presence and absence of an electrical double layer force in order to investigate the effect of surface charge density on the magnetic core-shell particle interactions. For core sizes of 0.5 and 4.0 μm the relative shell thicknesses needed to hinder the aggregation process were approximately 0.4 and 0.6 respectively, indicating that larger core sizes are detrimental to be used in applications in which no flocculation is needed. In addition, the presence of an electrical double layer, for values of surface charge density of less than 20 mC/m^2^, could stop the contact between particles without hindering their vertical alignment. Only when the shell thickness was considerably larger, was the electrical double layer able to contribute to the full disruption of the magnetic flocculation process.

## Introduction

Core-shell particles are particles whose cores are made of different component materials to those of the shell surrounding them (Cao et al., 2009; Mora-Huertas et al., 2010). In general, there is an inorganic or organic particle core which is surrounded by a layer or multilayer of a different inorganic or organic material. These core-shell particles have the advantage of possessing properties of both the core and its surrounding shell. This not only gives benefits in terms of improving the stability and surface chemistry of the core particle but also gives unique physical and chemical properties that are impossible to have if only one type of material is used (Caruso et al., 1999; Cao et al., 2009).

The structure of core-shell particles may be comprised of the core being a particle and the shell consisting of a different solid material. However, the shell could also be a soft layer of attached molecules, such as functional polymers which can be used to tune the specific surface interactions of the particles (Vakurov et al., 2009; Mora-Huertas et al., 2010; Yuan et al., 2010; Amelia et al., 2011; Arsianti et al., 2011). Core-shell particles are fabricated via a range of methods including dispersion polymerization, self-assembly techniques (Cao et al., 2009) and microfluidic jet-spray drying (Amelia et al., 2012). The properties of the hybrid multifunctional particles formed can be controlled by altering the composition, dimension and structure of the cores and the shells.

Core-shell particles possess a high level of complexity in terms of structure and behavior under external forces including mechanical interaction with other particles (Centi and Perathoner, 2011; Sacanna and Pine, 2011; Strydom et al., 2014). It is quite common that either the core or the shell are made of a magnetic material (Zeng et al., 2004a,b) and these suspensions are known as magnetic or magnetorheological fluids (Bossis et al., 2011). Nevertheless, a larger degree of sophistication has been achieved by creating magnetic patches (Sacanna et al., 2012) or controlling shell thickness (Strydom et al., 2014).

Traditionally, magnetorheological fluids are suspensions of micron sized or smaller, magnetizable particles in a liquid (Vekas et al., 2000; Tao, 2001; Lopez-Lopez et al., 2005; Patel and Chudasama, 2009; Bossis et al., 2011; de Vicente et al., 2011). A special case of these magnetic fluids are ferrofluids in which the particles are ferromagnetic materials at the nanoscale (Rosensweig, 1985, 2011; Popplewell et al., 1995; Odenbach, 2009). These fluids are of interest to researchers and engineers due to their unique properties, since under an applied magnetic field their viscosity and other physical properties can be altered by orders of magnitude, depending on the intensity of the field (Bossis et al., 2011; de Vicente et al., 2011). At a more fundamental level, one of the main areas of research on magnetorheological fluids is the formation of self-organized structures, whether experimentally or using computer simulations, under the influence of an external magnetic field (Wang et al., 2002; Li et al., 2005; Han et al., 2010; Lim and Feng, 2012; Ku et al., 2015; Lagger et al., 2015a,b). Different interactions may lead to the formation of several types of structures, including linear chains, which align parallel to the external magnetic field (Parker et al., 1982; Perez-Castillo et al., 2000; Vekas et al., 2000; Martinez-Pedrero et al., 2007a; Mousavi et al., 2015). However, in applications such as drug delivery when either functionalized magnetic or core-shell particles are introduced into the blood system, the formation of magnetic chains is highly undesirable (Freund and Shapiro, 2012).

Computer simulations and theoretical studies have mainly focused on understanding particle aggregation (Hovorka et al., 2005; Zubarev et al., 2010) and rheological behavior (Chirikov et al., 2010; Kang et al., 2012; Segovia-Gutierrez et al., 2013) in constant or oscillating magnetic fields (Li et al., 2012). The trajectory of magnetic particles in a magnetic gradient (Li et al., 2007; van Netten et al., 2013), the influence of particle density on separation efficiency (Murariu et al., 2005) and simply the demonstration of the separation concept using model systems (Moeser et al., 2004; Nakai et al., 2011) has also been studied.

Simulation of core-shell particles has been performed by using molecular dynamics (MD) to study the actual properties of the particles but not their bulk behavior in suspension (Dalgic, 2016; Wang and Shin, 2017). It is only in recent years that a technique such as Discrete Element Method (DEM) has been applied to colloidal suspensions (Li et al., 2003; Moreno-Atanasio et al., 2009; MacPherson et al., 2012) and core-shell particles (Moreno-Atanasio et al., 2009; Weber et al., 2017). The main difference between DEM and MD is that the latter focuses on the analysis of molecules (Mijajlovic and Biggs, 2007) and the former, DEM, on the behavior of single particles (mesoscopic) whose material properties have been defined. DEM is easily coupled with Brownian dynamics to simulate the hydrodynamic interactions based on the Langevin equation (Li et al., 2003; Moreno-Atanasio et al., 2009). The main advantage of DEM is that the treatment of the bulk material (such as a particle suspension) is mesoscopic and the dimensions of the simulated systems are more realistic than in molecular dynamics. In addition, DEM simulations usually employ the material properties of the whole particle to analyze the contact between structures and the interaction which is provided by an external shell or coating as determined by atomic force microscopy (AFM) or by atomistic or molecular simulations (Patrick et al., 2003; Bose et al., 2005).

Despite the wide use of DEM in studying colloidal systems, the literature on using DEM to study magnetic colloidal systems is still incipient. Golovnia et al. (2014) studied the alignment of the magnetic particles with the external field. However, other published DEM studies considered the particles to be aligned along the external magnetic field. Zhenghua et al. (2010) investigated particle motion in a fluidization assisted by a magnetic gradient and they observed the fluidization at high values of external magnetic strength. Similar observations were reported by Wang et al. (2013). Lindner et al. (2013) combined DEM with FEM to determine the external magnetic field and the fluid flow around cylindrical wires. Other DEM studies focused on predicting the magnetic particle behavior during compression, shearing and breakage of magnetic chains were published by Kargulewicz et al. (2012); Lagger et al. (2015a,b); Soda et al. (2015). Ke et al. (2017) coupled DEM with Lattice Boltzmann Method and Immersed Boundary Method to simulate the settling of two individual magnetic particles. In addition, Son (2018) studied the hindered settling of magnetic particles coated with a surfactant layer, thus increasing the effective hydrodynamic radius of the particles. Despite the abovemetioned studies, the actual bulk behavior of magnetic core-shell colloidal particles has never been studied by considering the shell explicitly.

This paper presents the first DEM study of the behavior of magnetic core-shell particles where the shell and core components were independently considered in the simulations rather than just averaging the core and shell of the core-shell particles. In this study the particle shells were simulated as silica. Ferromagnetic cores of different sizes (0.5, 1.0, 2.0, and 4.0 μm) were simulated with a large overall particle size range of core-shell particles (0.6–21 μm) in order to vary the thickness of the shell layer and study its effect on chain formation. In addition, core-shell particles were simulated with different shell thicknesses and particle sizes in the presence and absence of an electrical double layer force in order to investigate the effect of surface charge density on the magnetic core-shell particle interactions.

## Methodology: DEM computer simulation optimization of coated iron particles

### Theory

Discrete Element Method (DEM) is a computational technique in which particles are assigned individual physical and mechanical properties. Although initially developed for the study of rock mechanics (Cundall and Strack, 1979) this technique has also been used to investigate the behavior of colloidal suspensions, especially in flocculation (Zhu and Yu, 2006; An et al., 2008) and in other colloidal systems (Moreno-Atanasio et al., 2009; Fang et al., 2012).

DEM uses Newton's second law to simulate particle motion. The equation of motion is integrated numerically using an explicit finite difference method, such as the Euler method (Cundall and Strack, 1979), to determine the accelerations, velocities and positions of the particles at any time. More detail on the DEM working procedure can be found elsewhere (MacPherson et al., 2012). The forces acting on the particles can be decomposed into several components: elastic contact, magnetic, electric double layer, Brownian and drag. Therefore, the general equation of motion can be written as:

(1) $$\begin{array}{l} m\frac{d^{2}\vec{x}}{dt^{2}}={\vec{F}}_{cont}+{\vec{F}}_{mag}+{\vec{F}}_{edl}+{\vec{F}}_{brw}+{\vec{F}}_{drag} \end{array}$$

where *m* is the mass of the particle, $\vec{x}$is the vector position of the particle, *t* the time, $\vec{F}$the force and the subscripts *cont, mag*, edl, *brw* and *drag* refer to contact, magnetic, electric double layer, Brownian and drag forces respectively.

The elastic force experienced by two contacting particles is given by Hertz's law:

(2) F→cont,n=43E*R*12δn32

where δ_*n*_ is the normal contact deformation, R^*^ is the reduced particle radius (1/R^*^= 1/R_1_ + *1/R*_2_), and E^*^ the reduced elastic modulus (1/E^*^= (1-$\upsilon_{1}^{2}$*)/E*_1_ + *(1-*$\upsilon_{2}^{2}$*)/E*_2_), of two contacting particles, respectively, as given by their individual radii, *R*_*i*_, Poisson's ratio, υ_*i*_ and elastic moduli, *E*.

The fluid in these simulations was assumed to be stationary. The Stokes' drag force for a stationary fluid used in the simulations is given by:

(3) $$\begin{array}{l} {\vec{F}}_{drag}=6\pi \nu R\left(\frac{d\vec{r}}{dt}\right) \end{array}$$

where ν is the fluid viscosity.

Brownian forces describe the random motion of particles in a fluid. The direction is randomly determined and the magnitude of the force follows a Gaussian, or normal distribution, with variance (< Δ${\text{F}}_{\text{B}}^{2}$ >), for a given direction (x, y, or z) in the form (MacPherson et al., 2012):

(4) $$\begin{array}{l} <\Delta F_{B}^{2}>=\frac{12K_{B}T\pi R\nu }{\Delta t} \end{array}$$

Where *k*_*B*_ is the Boltzmann constant, *T* the absolute temperature and Δ*t* the time step.

The electrical double layer force (*F*_*edl*_) was incorporated into the simulations by using the Debye-Huckel approximation for constant surface charge density in the form (Warren, 2000; Li et al., 2010; Szilagyi et al., 2014):

(5) $$\begin{array}{l} {\vec{F}}_{EDL}=\frac{q_{i}q_{j}}{4\pi \varepsilon_{0}\varepsilon_{r}r^{3}}exp^{-\left(r-\left(a+b\right)\right)\kappa }\frac{1+r\kappa }{\left(1+a\kappa \right)\left(1+b\kappa \right)}\vec{r} \end{array}$$

Where κ^−1^ is the Debye length, *a* and *b* are the particle radii of particles *i* and *j* respectively, *d* is the particle center to particle center distance, *r* is the distance between charges, ε_0_ is the permittivity of free space, ε_*R*_ is the relative dielectric constant and *q*_*i*_ and *q*_*j*_are the total surface charge for particles *i* and *j* respectively.

The magnetic force between two particles (*i* and *j*) was calculated as a function of the initial magnetic susceptibility (χ) of iron whose value is equal to 150. The expression used for the force between two magnetic dipoles (*F*_*m*_*)* was (Rosensweig, 1985; Patel and Chudasama, 2009; Lim and Feng, 2012; Ku et al., 2016):

(6) $$\begin{array}{l} {\vec{F}}_{m}=\frac{3\mu_{0}}{4\pi r^{5}}\left[\left({\vec{m}}_{i}\vec{r}\right){\vec{m}}_{j}+\left({\vec{m}}_{j}\vec{r}\right){\vec{m}}_{i}+\left({\vec{m}}_{i}{\vec{m}}_{j}\right)\vec{r}-\frac{5\left({\vec{m}}_{i}\vec{r}\right)\left({\vec{m}}_{j}\vec{r}\right)}{r^{2}}\vec{r}\right] \end{array}$$

where ${\vec{m}}_{i}$ and ${\vec{m}}_{j}$ are the magnetic dipolar moment of the particles *i* and *j* and ${\vec{r}}_{ij}$ is the vector that joins the center of particle *i* with the center of particle of *j*.

### Simulation details

A summary of the particle properties used in the simulations is given in Table 1. Particles were randomly positioned within a cubic space whose dimensions were varied in order to keep the volume fraction constant at the value of 0.0039, which is close to published experimental and simulation work (Ezzaier et al., 2017; Hyde et al., 2017). No overlap between the particles was allowed in the initial configuration and no initial velocity was given to the particles. Therefore, particle motion was a direct consequence of the magnetic, van der Waals forces and Brownian forces.

**Table 1:** Particle physical properties and fluid properties.

**Property**	**Value**
Core density (kg/m^3^)	7,860
Shell density (kg/m^3^)	2,200
Elastic modulus–shell (GPa)	70
Magnetization (A/m)	1.8 × 10^5^
Volume fraction (v/v)	0.004
Water viscosity (Pa s)	0.001

The cores and shells were simulated as made of pure iron and silica respectively (Neville and Moreno-Atanasio, 2012; Hyde et al., 2017). The magnetization of the particles was set at 1/10^th^ of the saturation magnetization value of iron 0.228 T/μ_0_ (Reitz et al., 2008; Kargulewicz et al., 2012).

#### Absence of electrical double layer forces

The first study was conducted in the absence of an electrical double layer force with the objective of understanding the influence of the magnetic force on particle aggregation. In addition, these cases can be considered as the limiting case of a situation with an electrical double layer force but high ionic strength and therefore would constitute a special case of our second study which was carried out in the presence of an electrical double layer interaction.

Particle sizes ranged between 0.5 and 21 μm while the core diameters considered were 0.5, 1.0, 2.0, and 4 μm (Table 2). Table 2 also shows the relative shell thickness, RST, which is defined as:

(7) $$\begin{array}{l} RST=\frac{d_{p}-d_{c}}{d_{p}} \end{array}$$

where d_p_ and d_c_ are the particle and core diameters. Eq. (7) can also be interpreted as a function of the particle and core radii, *r*_*p*_ and *r*_*c*_, as:

(8) $$\begin{array}{l} RST=\frac{r_{p}-r_{c}}{r_{p}} \end{array}$$

**Table 2:** Properties of the different core-shell particles studied at the limit of the range for each core diameter.

**Simulation label**	**Particle diameter, d_p_, (μm)**	**Core diameter, d_c_ (μm)**	**Relative shell thickness**	**Concentration (g/l)**
05-C05	0.50	0.50	0.00	29.9
20-C05	2.00	0.50	0.75	8.60
10-C10	1.00	1.00	0.00	29.9
40-C10	4.00	1.00	0.75	8.60
20-C20	2.00	2.00	0.00	29.9
120-C20	12.0	2.00	0.83	8.40
40-C40	4.00	4.00	0.00	29.9
210-C40	15.0	4.00	0.81	8.50

It is important to note that the difference between particle radius and core radius, *r*_*p*_–*r*_*c*_, is the actual thickness of the shell. Both, Equations (7, 8) can take values between 0 and 1 which correspond to the cases of a particle with no shell and a particle in which there is no magnetic core, respectively.

Each simulation was labeled in terms of particle and core sizes and thus, the two first numbers represent the particle size in units of 100 nm and the two numbers following the letter C represent the core size, also in units of 100 nm. For example, case 06-C05 has a central core of 500 nm diameter (“C05”), with a 50 nm shell layer, giving an overall particle diameter of 600 nm (“06”). Therefore, the relative shell thickness, RST, would be according to Equations (7) or (8), 1/6 or 0.17.

#### In the presence of EDL forces

The first part of the analysis of the influence of the electrical double layer was carried out by varying the surface charge density. Surface charge densities in the range of 0.1 to 20 mC/m^2^ were assigned to the particles while the Debye length was kept constant at 3 nm (equivalent to a 10 mM monovalent salt solution). These surface charge densities correspond to surface potentials of 0.42–84 mV. In the case of unmodified silica, these values would be negative (Hyde et al., 2015). However, if the silica formed contained a basic polymer, the surface potentials would be positive, but of the same magnitude (Neville et al., 2013; Hyde et al., 2015). Therefore, the sign of the surface charge is irrelevant in this study as all the particles were considered to have the same sign of the surface potential and therefore, they will always experience repulsion due to the electrical double layer.

In order to elucidate if either the absolute or relative shell thickness are the most important parameters controlling aggregation, our study in the presence of electrical double layer considered the cases of a shell thickness of 50 nm with core sizes of 0.5 and 4 μm in diameter (cases 06-C05 and 41-C40), respectively. In order to compare the effect of relative shell thickness, RST, two cases with relative shell thickness equal to 0.17 and 0.64 for two different core diameters 0.5 and 4 μm were investigated. Here, the four cases were 06-C05 (RST = 0.17), 48-C40 (RST = 0.17), 15-C05 (RST = 0.64) and 120-C40 (RST = 0.64). Here the coordination number (number of neighbors per particle), singlet ratio (single particles/total particles) and largest fragment ratio (chain) (particles in largest fragment/total particles) were analyzed. After this, the minimum interparticle distance between particles that showed no contacts was analyzed. In addition, the minimum surface charge density for the particles to not be in contact was studied in order to obtain the relationship between this value and the ratio of the core to total particle diameter.

## Results and discussion

The focus of this paper was to investigate the influence of the shell thickness of magnetic core-silica shell particles on their colloidal stability, for different values of surface charge density. The properties of the cores and the shells were explicitly considered rather than the use of an overall average of the particle. Four different core sizes were simulated with a large overall particle size range (Table 2).

### Magnetic aggregation in the absence of electrical double layer (high ionic strength)

**Figure 1:**
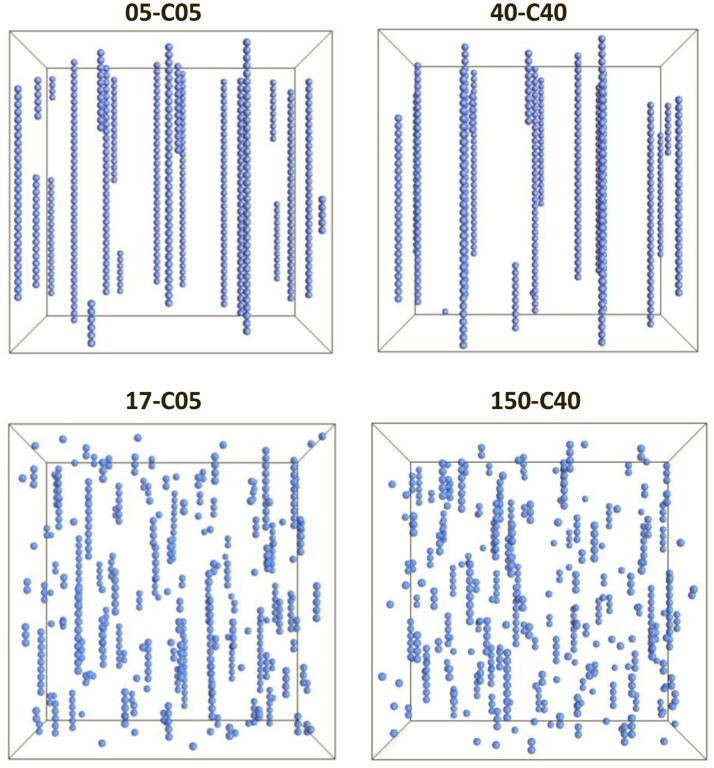
Visualization of 4 cases corresponding to two different core sizes (0.5 and 4.0 μm, left and right respectively) and situations in which no shell was present, and thus RST = 0 (top) or a thick shell was present (RST = 0.70, bottom left and RST = 0.73, bottom right). The images have been scaled for easier viewing. However, the dimensions of the system were different to keep a constant volume fraction of 0.0039.

All the simulations conducted here used the properties given in Tables 1, 2. Figure 1 shows the visualization of the system for the cases in which the relative shell thickness (RST) were 0.0 (top), and 0.70 and 0.73 (left and right, bottom respectively).

The simulations in Figure 1 clearly demonstrate that the presence of a shell (Figure 1, bottom) can disrupt the chain formation process that can be seen when the particles had no shell (Figure 1, top). In fact, although some chains were formed in the presence of a thick shell, their linearity as well as their length seem to be greatly affected. Furthermore, in the absence of a shell, the chains seem to transverse the system from top to bottom as typically encountered in magnetic fluids studies (Popplewell and Rosensweig, 1996; Chin et al., 2000). However, the case 40-C40 shows a smaller number of chains which seem to be longer than in the 05-C05 case, corroborating that larger magnetic particles aggregate more easily. However, a microscopic analysis is necessary in order to understand the influence of the presence of a shell and the differences between the different cases.

**Figure 2:**
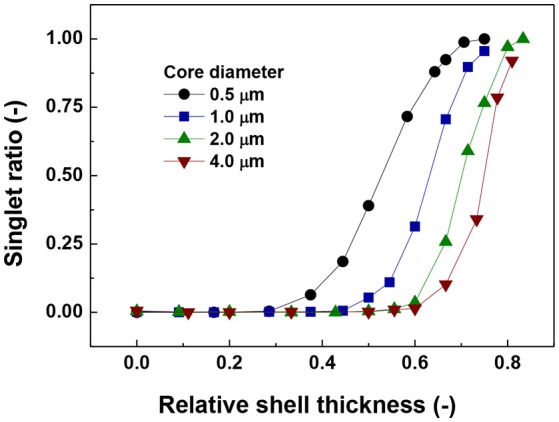
Ratio of number of singlets to the total number of particles vs. relative shell thickness.

The data in Figure 2 show the singlet ratio (number of particles without contacts divided by the total number of particles) against the relative shell thickness for different core sizes. The general trend for all core diameters is that the ratio of the singlets increases with the increase in relative shell thickness. In addition, the actual size of the core has a strong influence on the behavior of the system as a magnetic core of 4.0 μm would need a shell thickness of around 6.0 μm to start to hinder aggregation as reflected by the increase in singlet ratio, while for the smallest core (0.5 μm) the required shell thickness is 0.46 μm.

**Figure 3:**
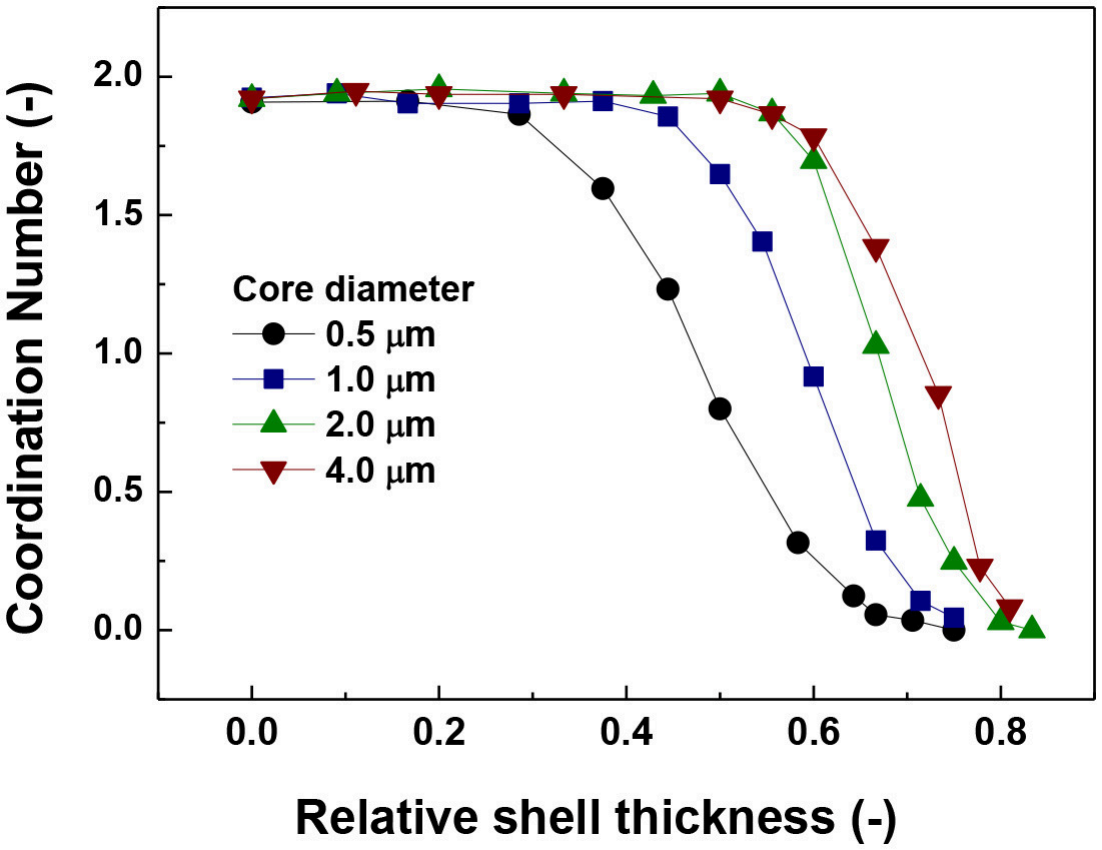
Coordination number vs. relative shell thickness.

A clearer picture is provided in Figure 3 where the coordination number as a function of the relative shell thickness is presented. Coordination number is defined as twice the number of contacts divided by the number of particles. The reason for the factor of two is that a contact is shared by two particles. It can be seen that a coordination number of around 2 was observed for low values of relative shell thickness, indicating that each particle has two contacts. This is indicative of the formation of linear chains due to the magnetic force and this coordination number is clearly much smaller than in self-assembly cases due van der Waals force (MacPherson et al., 2012). It can also be observed that the coordination number decreased with the increase in relative shell thickness, suggesting the occurrence of disruption of the chain formation process. Finally, the increase in the actual value of the size of the core is associated to an increase in the coordination number and thus, this observation confirms that the larger the size of the core, the largest the relative shell thickness needed to disrupt the magnetic aggregation.

**Figure 4:**
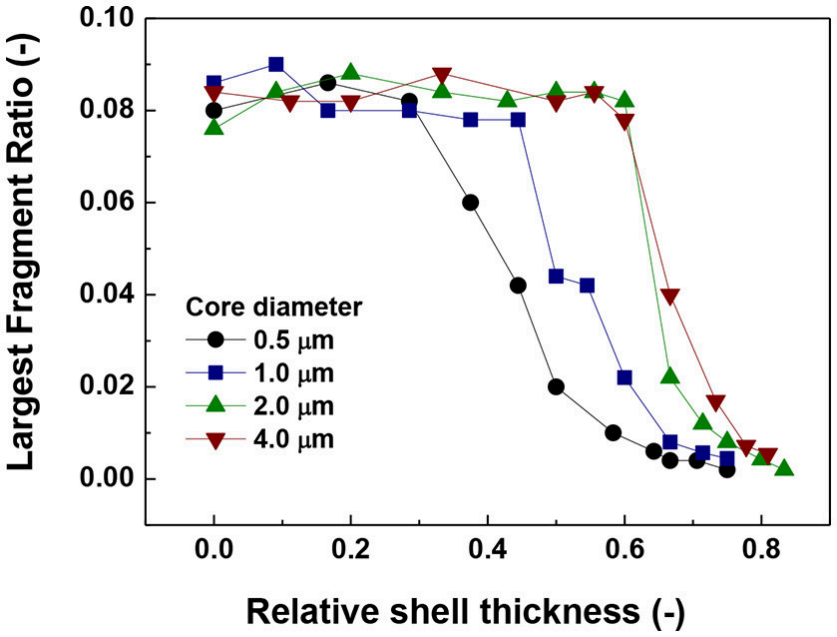
Largest fragment ratio vs. relative shell thickness.

In order, to fully understand the degree of aggregation, we have plotted the largest fragment ratio (number of particles contained in the largest fragment divided by the number of particles in the system) a function of the relative shell thickness in Figure 4. As can be seen in Figure 4, there is a decrease in the size of the largest fragment with increasing relative shell thickness (RST). All the curves trend to around 0.08 for the smallest values of RST. This value of the largest fragment ratio although seemingly small, corresponds to an actual length of 40 particles which can be easily demonstrated to coincide with the length of the working space, L /d_p_ is

(9) $$\begin{array}{l} \frac{L}{d_{p}}={\left(\frac{\pi N}{6\phi }\right)}^{1/3} \end{array}$$

where N is the number of particles in the system and φ is solid volume fraction (Table 1). As the number of particles in the system as well as the packing were fixed for all the simulations at 500 and 0.0039, L/d_p_ is equal to 40.6 particles.

The value of 0.08 starts to decrease approximately at the same relative shell thickness value at which the coordination number started to decrease (Figure 2) and the number of singlets started to increase (Figure 1). The coincidence of these points suggests a predominant behavior of single particle addition rather than cluster-cluster aggregation.

Another important aspect is the fluctuation that appears in the curves of the largest fragment (Figure 4) in comparison to the singlets and coordination number plots (Figures 2, 3). This is more likely to be due to a statistical process in which the random nature of the Brownian forces produces aggregates of slightly different sizes. This result is consistent with the trend of coordination number and number of singlets suggesting that there is a maximum value of shell thickness below which the presence of the shell does not have an important influence on the behavior of the system. Nevertheless, it is important to note that this maximum value increases with increasing core size.

### In the presence of an electrical double layer contribution

After the initial study where no electrical double layer contribution was included, chain formation in the presence of an electrical double layer force was investigated in order to determine the surface charge density required to act on the shell layer for particle contacts not to occur in chains. The effect of the electrical double layer on particles with different shell thicknesses was also studied to determine magnetic core-shell particle interaction behavior.

**Figure 5:**
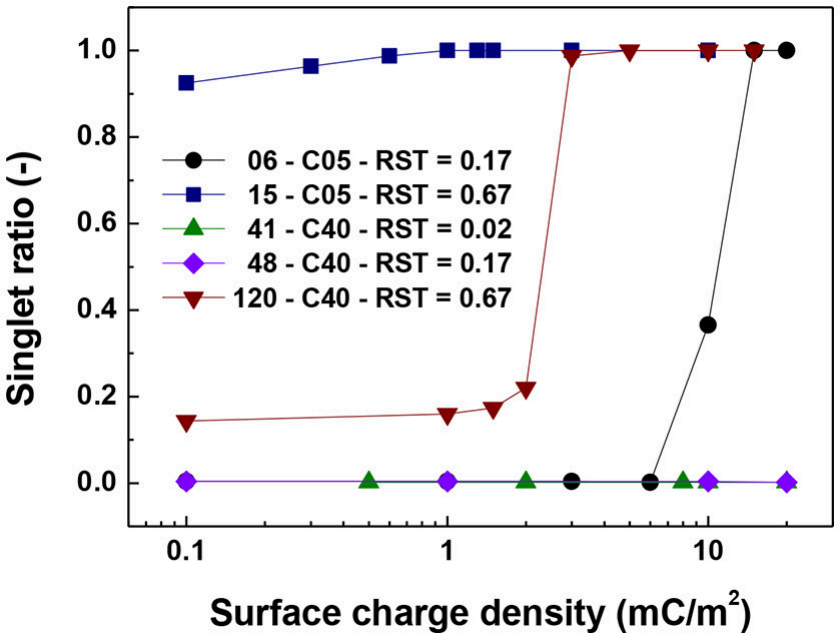
Singlet ratio vs. surface charge density. The figure presents the comparison for cases of shell thickness equal to 50 nm (06-C05 and 41-C40) and two values of relative shell thickness of 0.17 (06-C05, 48-C40) and 0.67 (15-C05 and 120-C40).

**Figure 6:**
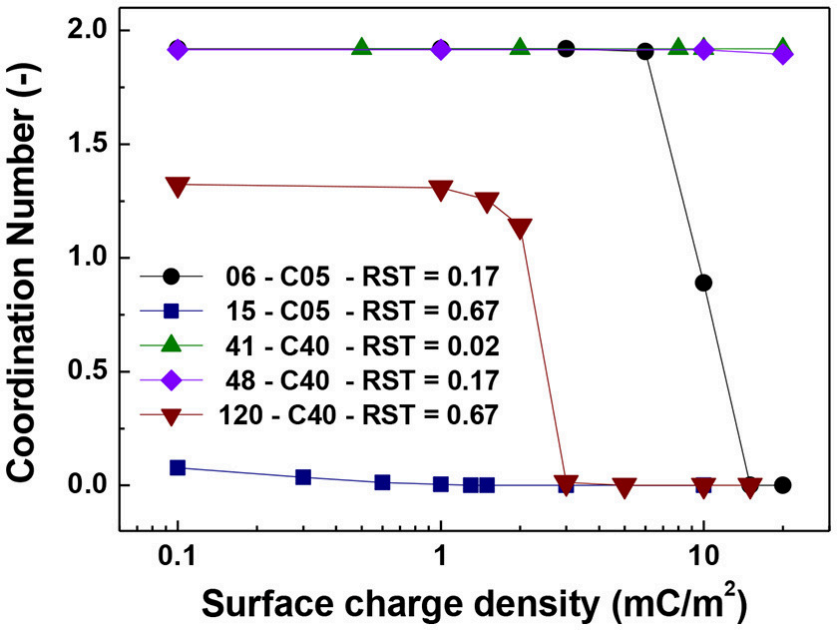
Coordination number vs. surface charge density. The figure presents the comparison for cases of shell thickness equal to 50 nm (06-C05 and 41-C40) and two values of relative shell thickness of 0.17 (06-C05, 48-C40) and 0.67 (15-C05 and 120-C40).

**Figure 7:**
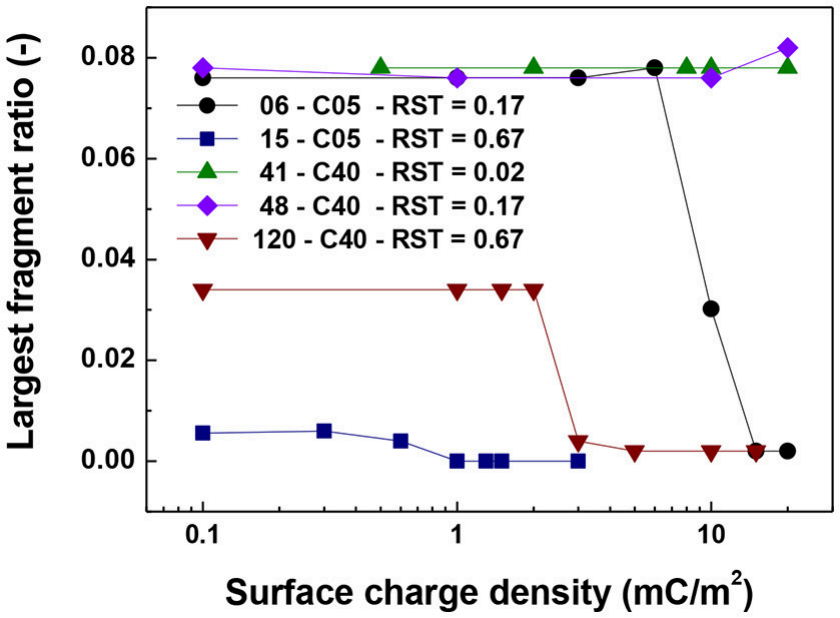
Largest fragment ratio vs. surface charge density. The figure presents the comparison for cases of shell thickness equal to 50 nm (06-C05 and 41-C40) and two values of relative shell thickness of 0.17 (06-C05, 48-C40) and 0.67 (15-C05 and 120-C40).

Figures 5–**7** show the singlet ratio, coordination number and largest fragment ratio as a function of the surface charge density for the five different cases considered in the simulations. For the smallest core (0.5 μm) there is a drastic difference between both plotted cases correlating well with the strong difference in shell thicknesses that are 50 nm and 500 nm. For the smallest particles (06-C05) a surface charge density of about 10 mC/m^2^ was required to start to observe the hindering of the magnetic aggregation. In contrast, for the largest shell thickness (15-C05) the magnetic core was unable to produce aggregation. This result is consistent with Figure 6 where we observed that the coordination number of the system is close to 2 for the former case and close to zero for the latter.

For the cases in which a 4.0 μm core size was simulated, the differences were not as abrupt as for the case of 0.5 μm core diameter. Figure 5 shows that the singlet ratio was close to zero or in the case of the largest relative shell thickness (0.67, case 120-C40) only about 20% of particles had no contacts, indicating that significant aggregation occurred. This is further corroborated by Figures 6, 7 as the coordination number and largest size ratio were close to 1.3 and 0.03, respectively, for the case 120-C40. Given that a relative shell thickness for the core of 0.5 μm was enough to completely hinder aggregation, we can conclude as the size of the core increases, even with the combined effect of the electric double layer repulsion and the thickness of the shell the possibility of stopping magnetic aggregation reduces considerably. This is a consequence of the magnetic force being proportional to the volume of the core (Equation 6) while the electrical double layer and the Brownian forces are proportional to the surface of the particles (Equation 4) and the square root the particle size (Equation 5), respectively.

By comparing the cases with a shell thickness of 50 nm (06-C05 and 41-C40), the presence of an electrical double layer was not sufficient to stop the aggregation process despite the magnetization was only 1/10th of the saturation magnetization of iron. The exception was observed for the smallest core and surface charge densities of 10 mC/m^2^ (42 mV). Therefore, within the range of particle size analyzed here the presence of a charged shell unless, further functionalized, has no role in the colloidal stability of the system.

**Figure 8:**
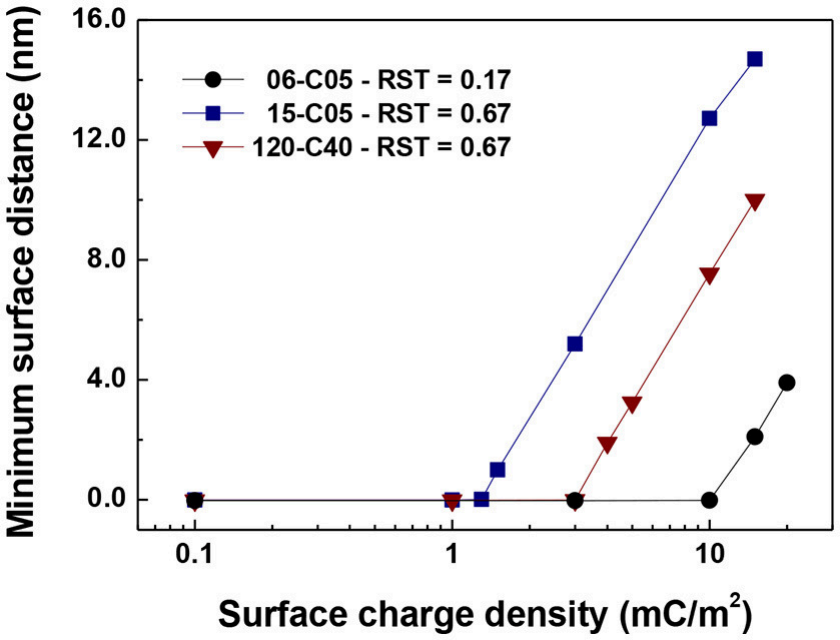
Minimum interparticle distance between particles for the cases plotted in Figures 5–7 that show no contacts (15-C05 and 120-C40 and 06-C05).

Figure 8 shows the minimum interparticle distance between particles for the cases plotted in Figures 5–7 that show no contacts for any value of surface charge density. These cases are 06-C05 at 15 mC/m^2^, 15-C05 at 3 mC/m^2^, and 120-C40 at 5 mC/m^2^. The values were obtained by averaging the minimum surface distance taken from the simulations between 0.2 and 1.0 s at 0.0001 s intervals. Figure 8 shows that the minimum surface distance increases “linearly” with surface charge density on the linear-log scale presented. This result is consistent with the experimental data of Chin et al. (2000) which observed that as zeta potential increased, the distance between particle increased with a natural logarithmic-shaped trend. They reasoned that this was because as the surface charge density increases the electrostatic repulsion increases and so the separation distance is larger (Chin et al., 2000). It is important to note that this minimum separation distance is orders of magnitude smaller than the particle diameter.

Most of the work published in the literature has focused on the aggregation of nanoparticles for which Brownian forces are mainly dominant. Chin et al. (2000) studied the superparamagnetic chain formation and breakage for particles of 0.89 μm diameter dispersed at different pH values and ionic strengths. Their suspension concentration was very dilute with 2.4 × 10^8^ particles/mL, which corresponds to a volume fraction of 8.9 × 10^−5^ (around 50 times smaller than ours). They first created the magnetic chains using magnetic field induction values in the range 0.5–0.8 T and once the chains were formed, the magnetic field induction was reduced to 0.0004 T. The latter value yields a magnetisation of 31.8 A/m and a magnetic moment for their particles of 1.17 × 10^−17^ Am^2^, which is significantly smaller than ours (9.5 × 10^−14^ Am^2^ and 1.2 × 10^−14^ Am^2^ for the 1 and 0.5 μm particles respectively). However, despite their much lower magnetization they still found formation of linear chains that were stable and did not break. Therefore, the chain formation and stability for the core-particles of 0.5 and 1.0 μm is consistent with the results of Chin et al. (2000).

Chin et al. (2000) studied the position of a secondary minimum for a range of magnetic field induction values and ionic strengths. They reported the position of the secondary minimum to be at 18 nm (surface to surface distance) for an ionic strength of 10 mM and 66 mV surface potential. This result (Chin et al., 2000) seems to be weakly independent of particle size as the value was very similar when either a 0.89 or 4 μm diameter particle was used. With this data the equivalent surface charge density calculated using

(10) $$\begin{array}{l} \sigma =\frac{\psi \varepsilon_{r}\varepsilon_{0}\left(1+\kappa R\right)}{R} \end{array}$$

is 15 mC/m^2^. Using their values of magnetic field induction of 0.05 T and magnetic susceptibility of 0.1, an equivalent magnetic moment of 1.47 × 10^−15^ Am^2^ is obtained.

Since the particles of Chin et al. (2000) are superparamagnetic composites of polystyrene and magnetic and our particles may be thought of as composite core-shell particles of silica and ferromagnetic iron, of similar but not identical sizes, a direct comparison is not straightforward. Nevertheless, according to Figure 8 for the same value of the electrical double layer, a separation distance 4–15 nm was obtained for the 0.5 μm core particles (with 0.6 and 1.5 μm total diameters), which is comparable to the value of Chin et al. (2000).

**Figure 9:**
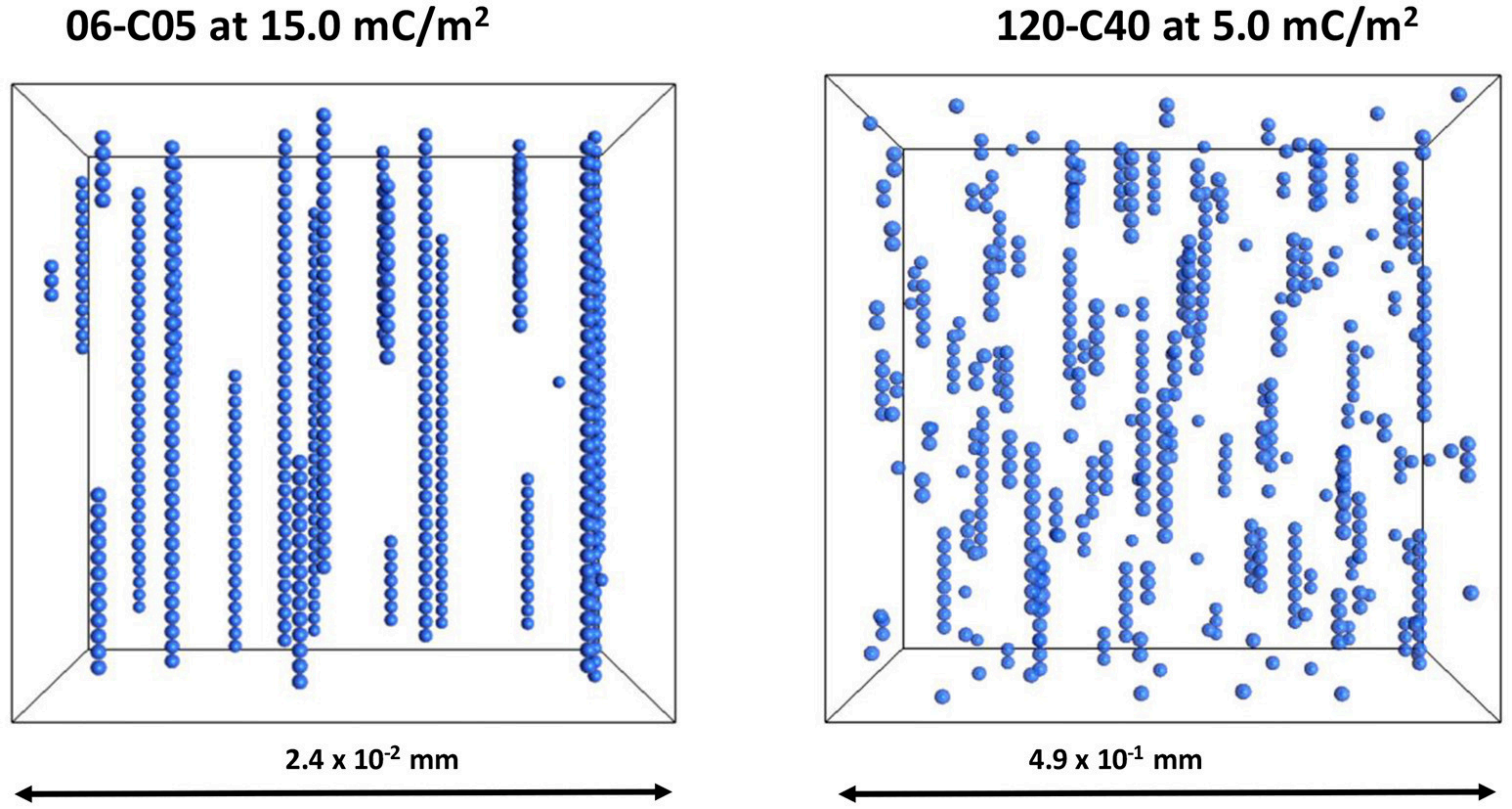
Visualization of the cases 06-C05 and 120-C40 at 15 and 5 mC/m^2^. These cases have relative shell thickness of 0.17 and 0.67. The images have been scaled for easier viewing. However, the dimensions of the system were different to keep a constant volume fraction of 0.0039.

A visualization of the 06-C05 and 120-C40 cases at 15 and 5 mC/m^2^ is provided in Figure 9. The differences in Figure 9 are remarkable as despite the fact that no contacts were detected in either case, there are still clearly visible linear particle chains, more so in the 06-C05 case than in the 120-C40. As shown in Figure 8 the minimum interparticle distance was much smaller than the particle size therefore, deceptively, the particles seem to be in contact in Figure 9 although this is not the case. More importantly, the level of disruption is much larger in the case of 120-C40, arguably due to the combined effect of shell thickness and electrical double layer. This is consistent with the results of Figures 2–4 where a RST of 0.67 started to affect chain formation in the absence of an electrical double layer. In contrast, an RST of 0.17 for a 0.5 μm core (06-C05 case), is too small to affect the aggregation process as this phenomenon would have required a RST of 0.35–0.40 (Figures 2–4) to start to observe the effects of its shell thickness in the case of 06-C05 (RST = 0.17). Therefore, chains seem to be forming without the particles being in physical contact in the 06-C05 cases as a consequence of the combination of magnetic and electrical double layer interactions having a primary (Gao et al., 2017) or a secondary minimum (Chin et al., 2000) outside the particle.

## Conclusions

Computer simulations based on the Discrete Element Method have been carried out to study the influence of shell thickness of magnetic core-silica shell particles. The investigation was carried assuming an arbitrary value of magnetization of the cores equivalent to one 10th of the saturation magnetization of iron (0.228/μ_0_).

The results have shown that the presence of a shell could contribute to the disruption of chain formation due to magnetic dipole-dipole interactions even in the absence of an electrical double layer. In addition, the increase in the size of the magnetic core produced an increase in the relative shell thickness needed to affect magnetic aggregation. This was attributed to the dependency on particle volume of the magnetic moments of the cores in comparison to the square root dependency of the Brownian forces and the physical presence of the shell whose influence could be considered to be linear with particle size.

The presence of an electrical double layer repulsion for particles of 0.5 μm core with a shell thickness of 50 nm was not sufficient to hinder the aggregation process until a surface charge density above 10 mC/m^2^ was achieved. In fact, although particles were shown not be in contact, it was possible to still observe the formation of linear chains with the particles probably positioned at a primary minimum located outside the particles. Nevertheless, the fact that particles were not in contact and thus fluid was present between the particles, suggests that a shear flow could easily disrupt the chain process and these particles could be useful in micro or nanofluidic devices. In contrast, when the shell thickness was sufficiently large to start to produce the disruption of the formation of linear chains, the electrical double layer repulsion could produce a total disruption of the magnetic aggregation.

This study suggests that it is necessary to tune the thickness of magnetic core-silica shell particles able to hinder the aggregation process in the absence of other steric repulsions (Almusallam and Sholl, 2007), as the electrical double layer was only effective when large shell thicknesses were considered (Martinez-Pedrero et al., 2007a,b; Baldasarre et al., 2015). Future work will include the incorporation of further parameters such as van der Waals force and the redefinition of contacts and singlets to include particles that are aligned in a chain formation, but not in physical contact.

## Author contributions

RM-A designed the computer code, ran simulations, and carried out a large amount of the analysis. FN also ran simulations using the code written by RM-A and obtained the additional results. RM-A and FN drafted the work. FN concentrated on the introduction and method sections and RMA on the results and discussion. RM-A and FN both corrected the full paper several times through.

### Conflict of interest statement

The authors declare that the research was conducted in the absence of any commercial or financial relationships that could be construed as a potential conflict of interest.

## References

[B1] Almusallam A. S. Sholl D. S. (2007). Brownian dynamics simulations of copolymer-stabilized nanoparticles in the presence of an oil–water interface. J. Colloid Interf. Sci, 313, 345–352. 10.1016/j.jcis.2007.04.017 17509608

[B2] Amelia R. Wu W. D. Cashion J. Bao P. Zheng R. Chen X. D. (2011). Microfluidic spray drying as a versatile assembly route of functional particles. Chem. Eng. Sci. 66, 5531–5540. 10.1016/j.ces.2011.07.059

[B3] Amelia R. Xu W. D. Chen Z. D. Selomulya C. (2012). Assembly of magnetic microcomposites from low pH precursors using a novel micro-fluidic-jet-spray-dryer. Chem. Eng. Res. Des. 90, 150–157. 10.1016/j.cherd.2011.06.014

[B4] An X. Z. Yang R. Y. Zou R. P. Yu A. B. (2008). Effect of vibration condition and inter-particle frictions on the packing of uniform spheres. Powder Technol. 188, 102–109 10.1016/j.powtec.2008.04.001

[B5] Arsianti M. Lim M. Lou S. N. Goon I. Y. Marquis C. P. Amal R. (2011). Bi-functional gold-coated magnetite composites with improved biocompatibility. J. Colloid Interf. Sci. 354, 536–545. 10.1016/j.jcis.2010.10.061 21131002

[B6] Baldasarre F. Cacciola M. Ciccarella G. J. (2015). A predictive model of iron oxide nanoparticles flocculation tuning Z-potential in aqueous environment for biological application. Nanopart. Res. 17:377 10.1007/s11051-015-3163-6

[B7] Bose K. Moreno R. Antony S. J. Ding Y. Biggs S. R. Ghadiri M. (2005). Effect of Contact Stiffness on the fluidization behaviour of cohesive powders, in Powder & Grains, eds García-Rojo R. Herrmann H. J. McNamara S. (London: CRC Press), 555–558.

[B8] Bossis G. Iskakova I. Kostenko V. Zubarev A. (2011). Statistical mechanics and its applications. Physica A 390, 2655–2663. 10.1016/j.physa.2011.02.044

[B9] Cao S. Chen J. Hu J. (2009). The fabrication and progress of core-shell composite materials Aust. J. Chem. 62, 1561–1576. 10.1071/CH08420

[B10] Caruso F. Susha A. S. Giersig M. Möhwald H. (1999). Magnetic core–shell particles: preparation of magnetite multilayers on polymer latex microspheres. Adv. Mater, 11, 950–953. 10.1002/(SICI)1521-4095(199908)11:11<950::AID-ADMA950>3.0.CO;2-T

[B11] Centi G. Perathoner S. (2011). Creating and mastering nano-objects to design advanced catalytic materials. Coordin. Chem. Rev., 255, 1480–1498. 10.1016/j.ccr.2011.01.021

[B12] Chin C. J. Yiacoumi S. Tsouris C. Relle S. Grant S. B. (2000). Secondary-minimum aggregation of superparamagnetic colloidal particles. Langmuir 16, 3641–3650. 10.1021/la991201n

[B13] Chirikov D. N. Fedotov S. P. Iskakova L. Y. Zubarev A. Y. (2010). Viscoelastic properties of ferrofluids. Physic. Rev. E 82:51405. 10.1103/PhysRevE.82.051405 21230477

[B14] Cundall P. A. Strack O. D. L. (1979). A discrete numerical model for granular assemblies. Geotechnique 29, 47–65. 10.1680/geot.1979.29.1.47

[B15] Dalgic S. S. (2016). Size dependent properties of hollow gold nanoparticles: a theoretical investigation. Acta Phys. Polon. A 129, 531–534. 10.12693/APhysPolA.129.531

[B16] de Vicente J. Klingerberg D. J. Hidalgo-Alvarez R. (2011). Magnetorheological fluids: a review. Soft Matter 7, 3701–3710. 10.1039/c0sm01221a

[B17] Ezzaier H. Alves Marins J. Razvin I. Abba M. Ben Haj Amara A. Zubarev A.. (2017). Two-stage kinetics of field-induced aggregation of medium-sized magnetic nanoparticles. J. Chem. Phys. 146:114902 10.1063/1.4977993 28330343

[B18] Fang H. Tade M. O. Li Q. (2012). A numerical study on the role of geometry confinement and fluid flow in colloidal self-assembly. Powder Technol. 214, 283–291. 10.1016/j.powtec.2011.08.023

[B19] Freund J. B. Shapiro B. (2012). Transport of particles by magnetic forces and cellular blood flow in a model microvessel. Phys. Fluids 24:051904 10.1063/1.4718752

[B20] Gao Y. Evans G. M. Wanless E. J. Moreno-Atanasio R. (2017). DEM modelling of particle-bubble capture through extended DLVO theory. Colloids Surf. A Physicochem. Eng. Aspects 529, 876–885. 10.1016/j.colsurfa.2017.06.082

[B21] Golovnia O. A. Popov A. G. Sobolev A. N. Hadjipanayis G. C. (2014). Alignment of magnetic uniaxial particles in a magnetic field: simulation. J. Magn. Magn. Mater. 365, 64–69. 10.1016/j.jmmm.2014.04.037

[B22] Han K. Feng Y. T. Owen (2010) Three-dimensional modelling simulation of magnetorheological fluids. Int. J. Numer. Meth. Eng. 84 1273–1302. 10.1002/nme.2940

[B23] Hovorka O. Dan Y. N. Friedman G. (2005). Self-consistent model of field gradient driven particle aggregation in magnetic fluids. J. Appl. Phys. 97:10Q306. 10.1063/1.1860811

[B24] Hyde E. D. E. R. Moreno-Atanasio R. Neville F. (2017). Fabrication of magnetic core PEI-silica shell particles. Mater. Res. Bull. 96, 222–232. 10.1016/j.materresbull.2017.02.045

[B25] Hyde E. D. Moreno-Atanasio R. Millner P. A. Neville F. (2015). Surface charge control through the reversible adsorption of a biomimetic polymer on silica particles. J. Phys. Chem. B 119, 1726–1735 10.1021/jp5100439 25543459

[B26] Kang T. G. Julsen M. A. den Toonder M. J. (2012). Dynamics of magnetic chains in a shear flow under the influence of a uniform magnetic field. Phys. Fluids 24:42001 10.1063/1.4704822

[B27] Kargulewicz M. Iordanoff I. Marrero V. Tichy J. (2012). Modeling of magnetorheological fluids by the discrete element method. J. Tribol. 134:31706 10.1115/1.4006021

[B28] Ke C.-H. Shu S. Zhang H. Yuan H.-Z. (2017). LBM-IBM-DEM modelling of magnetic particles in a flui. Powder Technol. 314, 264–280. 10.1016/j.powtec.2016.08.008

[B29] Ku J. G. Liu X. Y. Chen H. H. Deng R. D. Yan Q. X. (2016). Interaction between two magnetic dipoles in a uniform magnetic field. AIP Adv. 6:25004 10.1063/1.4941750

[B30] Ku J. Chen H. He K. Yan Q. (2015). Simulation and observation of magnetic mineral particles aggregating into chains in a uniform magnetic field. Min. Eng. 79, 10–16. 10.1016/j.mineng.2015.05.002

[B31] Lagger H. G. Bierwisch C. Korvink J. G. Moseler M. (2015a). Discrete element study of viscous flow in magnetorheological fluids. Rheol. Acta 53, 417–443 10.1007/s00397-014-0768-0

[B32] Lagger H. G. Breinlinger T. Korvink J. G. Moseler M. Di Renzo A. Di Maio F. (2015b). Influence of hydrodynamic drag model on shear stress in the simulation of magnetorheological fluids. J. Non Newtonian Fluid Mech. 218:16026 10.1016/j.jnnfm.2015.01.010

[B33] Li D. Lam C. N. Biswal S. L. (2010). Measuring short-range repulsive forces by imaging directed magnetic-particle assembly title. Soft Matter 6, 239–242 10.1039/B917675F

[B34] Li H. Peng X. Chen W. (2005). Simulation of the chain-formation process in magnetic fields. J. Intell. Mater. Syst. Struct. 16, 653–658. 10.1177/1045389X05052598

[B35] Li J. Yu W. Chen C. Wei W. (2003). Modeling nanosized colloidal particle interactions with Brownian dynamics using discrete element method. 2003 Nanotechnology Conference and Trade Show. Nanotech 2, 566–569.

[B36] Li X. L. Yao K. L. Liu H. R. Liu Z. L. (2007). The investigation of capture behaviors of different shape magnetic sources in the high-gradient magnetic field. J. Magn. Magn. Mater. 311, 481–488. 10.1016/j.jmmm.2006.07.040

[B37] Li Y. H. Chen C. Y. Sheu S. T. Pai J. M. (2012). Dynamics of a microchain of superparamagnetic beads in an oscillating field. Microfluid Nanofluid 13, 579–588. 10.1007/s10404-012-0974-y

[B38] Lim E. W. Feng R. (2012). Agglomeration of magnetic nanoparticles. J. Chem. Phys. 136:124109. 10.1063/1.3697865 22462837

[B39] Lindner J. Menzel K. Nirschl H. (2013). Simulation of magnetic suspensions for HGMS using CFD, FEM and DEM modelling. Comp. Chem. Eng. 54, 111–121. 10.1016/j.compchemeng.2013.03.012

[B40] Lopez-Lopez M. T. de Vicente J. Bossis G. González-Caballero F. (2005). Preparation of stable magnetorheological fluids based on extremely bimodal iron–magnetite suspensions. J. Mat Res. 20, 874–881 10.1557/JMR.2005.0108

[B41] MacPherson S. A. Webber G. B. Moreno-Atanasio R. (2012). Aggregation of nanoparticles in high ionic strength suspensions: effect of Hamaker constant and particle concentration. *Adv*. Powder Technol. 23, 478–484 10.1016/j.apt.2012.04.008

[B42] Martínez-Pedrero F. Tirado-Miranda M. Schmitt A. Callejas-Fernández J. (2007a). Formation of magnetic filaments: a kinetic study. Phys Rev. E 76:11405. 10.1103/PhysRevE.76.011405 17677439

[B43] Martinez-Pedrero F. Tirado-Miranda M. Schmitt A. Vereda F. Callejas-Fernandez J. (2007b). Structure and stability of aggregates formed by electrical double-layered magnetic particles. Colloids Surf. A Physicochem. Eng. Aspects 306, 158–165. 10.1016/j.colsurfa.2007.03.029

[B44] Mijajlovic M. Biggs M. J. (2007). Study of conformational switching in polyalanine at solid surfaces using molecular simulation. J. Phys. Chem. C. 111, 15839–15847. 10.1021/jp074378w

[B45] Moeser G. D. Roach K. A. Green W. H. Hatton T. A. (2004). High-gradient magnetic separation of coated magnetic nanoparticles. AIChE J. 50, 2835–2848. 10.1002/aic.10270

[B46] Mora-Huertas C. E. Fessi H. Elaissari A. (2010). Polymer-based nanocapsules for drug delivery. Int. J. Pharm. 385, 113–142. 10.1016/j.ijpharm.2009.10.018 19825408

[B47] Moreno-Atanasio R. Antony S. J. Williams R. A. (2009). 'Influence of interparticle interactions on the kinetics of self-assembly and mechanical strength of nanoparticulate aggregates. Particuology 7, 106–113 10.1016/j.partic.2009.01.008

[B48] Mousavi N. S. Khapli S. D. Kumar S. (2015). Direct observations of field-induced assemblies in magnetite ferrofluids. J. Appl. Phys. 117:103907 10.1063/1.4914484 25829566 PMC4359171

[B49] Murariu V. Svoboda J. Sergeant P. (2005). The modelling of the separation process in a ferrohydrostatic separator. Miner. Eng. 18, 449–457. 10.1016/j.mineng.2004.06.015

[B50] Nakai Y. Senkawa K. Mishima F. Akiyama Y. Nishijima S. (2011). Study on interparticle interaction for dry HGMS system using pneumatic conveyance. Phys. C. 471:15331537 10.1016/j.physc.2011.05.232

[B51] Neville F. Moreno-Atanasio R. (2012). Magnetic interactions of core-shell composite particles: a combined experimental and simulation approach, in Chemeca 2012: Quality of Life through Chemical Engineering (Proceedings) (Wellington).

[B52] Neville F. Murphy T. Wanless E. (2013). The formation of polyethyleneimine-trimethoxymethylsilane organic-inorganic hybrid particles. Colloid. Surf. A Physicochem. Eng. Aspects 431, 42–50. 10.1016/j.colsurfa.2013.04.022

[B53] Odenbach S. (2009). Colloidal Magnetic Fluids: Basics, Development and Applications of Ferrofluids. Berlin: Springer.

[B54] Parker M. R. van Leef R. P. A. R. Myron H. W. Wyder P. (1982). Particle aggregation in colloids in high magnetic fields. J. Magm. Mag. Mat. 27, 250–256. 10.1016/0304-8853(82)90085-3

[B55] Patel R. Chudasama B. (2009). Hydrodynamics of chains in ferrofluid-based magnetorheological fluids under rotating magnetic field. Phys. Rev. E. 80:12401. 10.1103/PhysRevE.80.012401 19658750

[B56] Patrick D. L. Flanagan J. F. Kohl P. Lyden-Bell R. M. (2003). Atomistic molecular dynamics simulations of chemical force microscopy. J. Am. Chem. Soc. 125, 6762–6773. 10.1021/ja0345367 12769587

[B57] Perez-Castillo I. Perez-Madrid A. Tubi J. M. (2000). Chaining in magnetic colloids in the presence of flow. J. Chem. Phys. 113, 6443–6448. 10.1063/1.1308541

[B58] Popplewell J. Rosensweig R. E. (1996). Magnetorheological fluid composites. J. Phys. D Appl. Phys. 29, 2297–2303. 10.1088/0022-3727/29/9/011

[B59] Popplewell J. Rosensweig R. E. Siller J. K. (1995). Magnetorheology of ferrofluid composites. J. Magm. Mag. Mat. 149, 53–56. 10.1016/0304-8853(95)00336-3

[B60] Reitz J. R. Milford F. J. Christy R. W. (2008). Foundations of Electromagnetic Theory, 4th Edn. Boston: Addison-Wesley.

[B61] Rosensweig R. E. (1985). Ferrohydrodynamics. Cambridge: Cambridge University Press.

[B62] Rosensweig R. E. (2011). Towards ferrofluids with enhanced magnetization. J. Mag. Mag. Mater. 323, 1191–1197. 10.1016/j.jmmm.2010.11.004

[B63] Sacanna S. Pine D. J. (2011). Shape-anisotropic colloids: building blocks for complex assemblies. Curr. Op. Coll. Interf. Sci. 16, 96–105. 10.1016/j.cocis.2011.01.003

[B64] Sacanna S. Rossi L. Pine D. J. (2012). Magnetic click colloidal assembly. J. Am. Chem. Soc. 134, 6112–6115. 10.1021/ja301344n 22449143

[B65] Segovia-Gutierrez J. P. de Vicent J. Hidalgo-Alvarez R. Puertas A. M. (2013). Brownian dynamics simulations in magnetorheology and comparison with experiments. Soft Matt. 9, 6970–6977. 10.1039/c3sm00137g

[B66] Soda R. Takagi K. Ozaki K. (2015). Extended particle-based simulation for magnetic-aligned compaction of hard magnetic particles. J. Magn. Magn. Mater. 396, 128–134. 10.1016/j.jmmm.2015.08.018

[B67] Son K. J. (2018). A discrete element model for the influence of surfactants on sedimentation characteristics of magnetorheological fluids. Korea Aus. Rheol. J. 30, 29–29 10.1007/s13367-018-0004-z

[B68] Strydom S. Otto D. P. Stieger N. Aucamp M. E. Liebenberg W. de Villiers M. M. (2014). Self-assembled macromolecular nanocoatings to stabilize and control drug release from nanoparticles. Powder Technol. 256, 470–476. 10.1016/j.powtec.2014.01.088

[B69] Szilagyi I. Trefalt G. Tiraferri A. Maroni P. Borkovec M. (2014). Polyelectrolyte adsorption, interparticle forces, and colloidal aggregation. Soft Matter 10, 2469–2502. 10.1039/c3sm52132j 24647366

[B70] Tao R. (2001). Super-strong magnetorheological fluids. J. Phys. Cond. Matter 13, R979–R999. 10.1088/0953-8984/13/50/202

[B71] Vakurov A. Pchelintsev N. A. Forde J. Ó'Fágáin C. Gibson T. Millner P. (2009). The preparation of size-controlled functionalized polymeric nanoparticles in micelles. Nanotechology 20:295605. 10.1088/0957-4484/20/29/295605 19567946

[B72] van Netten K. Zhou J. Galvin K. P. Moreno-Atanasio R. (2013). ‘Influence of magnetic and hydrodynamic forces on chain-aggregation and motion of magnetisable particles and composites. Chem. Eng. Sci. 93, 229–237. 10.1016/j.ces.2013.01.028

[B73] Vékás L. Rasa M. Bica D. (2000). Physical Properties of Magnetic Fluids and Nanoparticles from Magnetic and Magneto-rheological measurements. J. Colloid Interface Sci. 231, 247–254. 10.1006/jcis.2000.7123 11049675

[B74] Wang J. Shin S. (2017). Room temperature nanojoining of Cu-Ag core-shell nanoparticles and nanowires. J. Nanopart. Res. 19:53 10.1007/s11051-017-3761-6

[B75] Wang S. Sun Z. Li X. Gao J. Lan X. Dong Q. (2013). Simulation of flow behavior of particles in liquid–solid fluidized bed with uniform magnetic field. Powder Technol. 237, 314–325. 10.1016/j.powtec.2012.12.013

[B76] Wang Z. Holm C. Müller H. W. (2002). Molecular dynamics study on the equilibrium magnetization properties and structure of ferrofluids. Phys. Rev. E. 66:21405. 10.1103/PhysRevE.66.021405 12241176

[B77] Warren P. B. (2000). A theory of void formation in charge-stabilized colloidal suspensions at low ionic strength. J. Chem. Phys. 112, 4683–4698 10.1063/1.481024

[B78] Weber M. Spettl A. Dosta M. Heinrich S. Schmidt V. (2017). Simulation-based investigation of core-shell agglomerates: influence of spatial heterogeneity in particle sizes on breakage characteristics. Comput. Mater. Sci. 137, 100–106. 10.1016/j.commatsci.2017.05.014

[B79] Yuan Q. Cayre O. J. Fujii S. Armes S. P. Williams R. A. Biggs S. (2010). Responsive core–shell latex particles as colloidosome microcapsule membranes. Langmuir 26, 18408–18414. 10.1021/la1033564 21028853

[B80] Zeng H. Li J. Wang Z. L. Liu J. P. Sun S. (2004a). Bimagnetic Core/Shell FePt/Fe3O4 Nanoparticles. Nano Lett. 4, 187–190. 10.1021/nl035004r

[B81] Zeng H. Sun S. Li J. Wand Z. L. Liu J. P. (2004b). Tailoring magnetic properties of core/shell nanoparticles. Appl. Phys. Lett. 85, 792–794. 10.1063/1.1776632

[B82] Zhenghua H. Xiang L. Huilin L. Guodong L. Yurong H. Shuai W. (2010). Numerical simulation of particle motion in a gradient magnetically assisted fluidized bed. Powder Technol. 203, 555–564. 10.1016/j.powtec.2010.06.022

[B83] Zhu H. P. Yu A. B. (2006). A theoretical analysis of the force models in discrete element method. Powder Technol. 161, 122–129. 10.1016/j.powtec.2005.09.006

[B84] Zubarev A. Y. Iskakova L. Y. Kostenko V. O. (2010). Kinetics of growth of chain aggregates in magnetic suspensions. Colloid J. 72, 799–805. 10.1134/S1061933X10060104

